# *IL10* Variant g.5311A Is Associated with Visceral Leishmaniasis in Indian Population

**DOI:** 10.1371/journal.pone.0124559

**Published:** 2015-05-05

**Authors:** Anshuman Mishra, Sheikh Nizamuddin, Geethika Arekatla, Satya Prakash, Hemlata Dewangan, Abishai Dominic, Abhishek Mishra, Digumarthi V. S. Sudhakar, Narasimha R. Parine, Nitin C. Tupperwar, Kumarasamy Thangaraj

**Affiliations:** 1 CSIR—Centre for Cellular and Molecular Biology, Hyderabad, India; 2 Oriental Institute of Science and Technology, Bhopal, India; University of Birmingham, UNITED KINGDOM

## Abstract

**Background:**

Visceral leishmaniasis (VL) is a multifactorial disease, where the host genetics play a significant role in determining the disease outcome. The immunological role of anti-inflammatory cytokine, Interleukin 10 (IL10), has been well-documented in parasite infections and considered as a key regulatory cytokine for VL. Although VL patients in India display high level of IL10 in blood serum, no genetic study has been conducted to assess the VL susceptibility / resistance. Therefore, the aim of this study is to investigate the role of *IL10* variations in Indian VL; and to estimate the distribution of disease associated allele in diverse Indian populations.

**Methodology:**

All the exons and exon-intron boundaries of IL10 were sequenced in 184 VL patients along with 172 ethnically matched controls from VL endemic region of India.

**Result and Discussion:**

Our analysis revealed four variations; rs1518111 (2195 A>G, intron), rs1554286 (2607 C>T, intron), rs3024496 (4976 T>C, 3’ UTR) and rs3024498 (5311 A>G, 3’ UTR). Of these, a variant g.5311A is significantly associated with VL (χ^2^=18.87; p =0.00001). In *silico* approaches have shown that a putative micro RNA binding site (miR-4321) is lost in rs3024498 mRNA. Further, analysis of the above four variations in 1138 individuals from 34 ethnic populations, representing different social and linguistic groups who are inhabited in different geographical regions of India, showed variable frequency. Interestingly, we have found, majority of the tribal populations have low frequency of VL (‘A’ of rs3024498); and high frequency of leprosy (‘T’ of rs1554286), and Behcet’s (‘A’ of rs1518111) associated alleles, whereas these were *vice versa* in castes. Our findings suggest that majority of tribal populations of India carry the protected / less severe allele against VL, while risk / more severe allele for leprosy and Behcet’s disease. This study has potential implications in counseling and management of VL and other infectious diseases.

## Introduction

Visceral leishmaniasis (VL), caused by protozoan parasite *Leishmania donovani*, is the most severe form of leishmaniasis. After infection, the parasite migrates to internal organs such as liver, spleen and bone marrow, followed by appearance of complex clinical symptoms, which can be lethal, if left untreated [1, 2]. In Indian subcontinent, (India, Nepal and Bangladesh) approximately 150 million people are at risk of developing VL (67% of the world VL disease) [3–5]. It is considered to be a rural disease and is a big burden for the people, who are in the villages of Bihar state in India [3, 6, 7]. Genetic, immunological and socio-economical factors play a role in the disease outcome [6–9].

**Fig 1:**
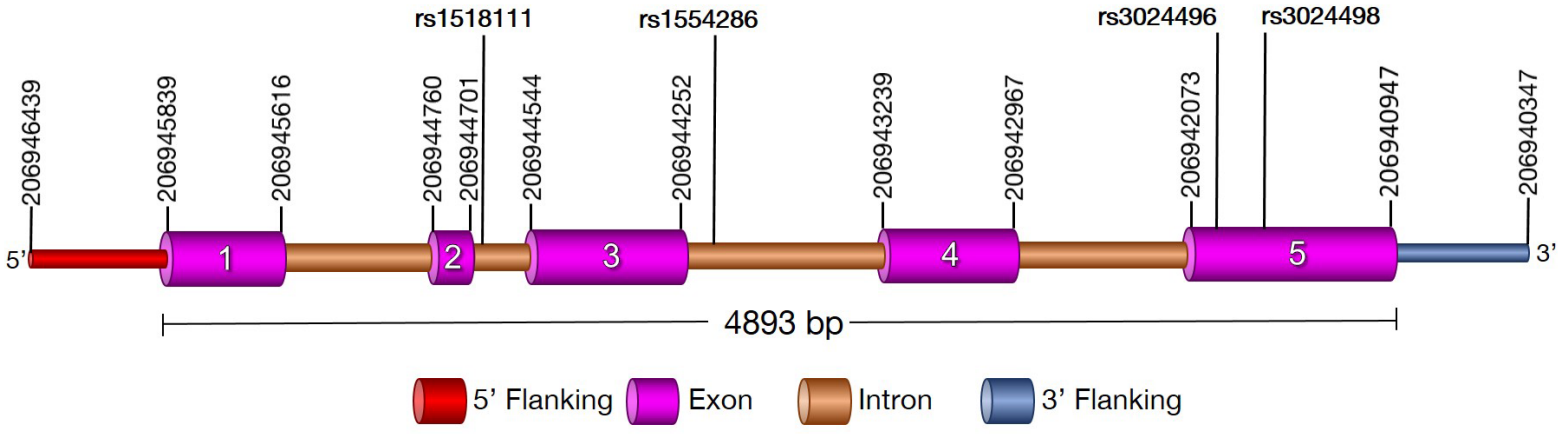
The structure of the human *IL10* (chr1, 206945839–206940947; ENST00000423557). Exons of the gene are shown in pink, introns in brown. rs1518111 (2195 A>G) and rs1554286 (2607 C>T) were the intronic variant of second and third exons while rs3024496 (4976 T>C) and rs3024498 (5311 A>G) were the 3’ UTR variant of fifth exons.

Human Interleukin-10 (IL10) gene, located on chromosomal region 1q32.1, codes for anti-inflammatory cytokine. IL10 comprises of 5 exons, covering approximately 4.8 kb (Fig 1). IL10 cytokine is primarily produced by monocytes and to a lesser extent by lymphocytes; namely type 2 T helper cells (Th2), mastocytes, CD4^+^CD25^+^Foxp3^+^ regulatory T cells, and a certain subset of activated T cells and B cells [10]. It is also expressed by different cells of the innate immune system, including dendritic cells (DCs), mast cells, natural killer (NK) cells, eosinophils and neutrophils [11]. IL10 down regulates the expression of Th1 cytokines, major histocompatibility complex II (MHC II), co-stimulatory molecules on macrophages and IL-12 [12,13]. IL10 has a stimulatory effect on certain T cells (Th2), mast cells and it stimulates the B cell survival, proliferation and antibody production [12, 14, 15]. It is also involved in the regulation of the STAT (Signal transducer and activator of transcription) signalling pathway and inhibit intracellular killing of amastigotes by macrophages [16, 17].

IL10 plays key role in different diseases, such as; hepatitis B, pulmonary tuberculosis, herpes zoster, cutaneous malignant melanoma, skin squamous cell carcinoma, inflammatory bowel diseases, human immuno deficiency viruses (HIV), leprosy, schistosomiasis, malaria, filaria and rheumatoid arthritis [18–29]. IL10 is also widely studied in organ transplantation [22, 30]. VL patients display over expression of IL10 mRNA and high level of IL10 in blood serum [10, 31] (reviewed in [12]). Recent studies on Indian VL demonstrated that disease outcome depends possibly on the balance between pro-inflammatory cytokines (IFN-γ and TNF-α) and anti-inflammatory (IL-10) responses [32, 33]. Subsequent studies have shown that the functional IL10 polymorphisms are also associated with pulmonary tuberculosis and leprosy in Indian population [19, 25]. Earlier genetic studies in Sudan, Brazil and Iran have shown the role of IL10 polymorphisms in visceral leishmaniasis (VL), cutaneous leishmaniasis (CL) and post kala-azar dermal leishmaniasis (PKDL) respectively [34–36]. However, to the best of our knowledge no attempt has been made to investigate the role of *IL10* in Indian VL. Therefore, we have investigated the complete *IL10* in ethnically matched VL case-controls. Considering the fact that, VL is endemic in Bihar state of India and every Indian population is genetically unique [37], we have also aimed to investigate the distribution of risk / protective / severe alleles, observed by the case-control study, among the 34 diverse population of India.

## Materials and Methods

### Sample collection

A total of 356 subjects, including 184 VL patients and 172 ethnically matched controls in the Middle Eastern part of India (Bihar state) were included in this study (Table 1). The sampling area were located within a radius of ~80 kilometer from the city of Muzaffarpur covering the districts of Muzaffarpur, Patna, Vaisali and Sitamadhi VL endemic regions. The demographic details of the study region and an annual incidence rate of 2.49 clinical VL cases/1,000 persons have been described elsewhere [38, 39].

**Table 1:** Sample size, mean age and sex ratio of case and control samples.

	Case (184)	Control (172)	P-value
Mean Age (year)+/-SD	29.38 +/- 17.11	38.79 +/- 16.57	0.33581
Male: Female	102:82	97:75	0.9397

Patients were recruited upon visiting their residence and screening their medical records, issued by the local government hospitals. Diagnosis of VL was performed at the hospitals by serological (rK39 strip test) and parasitological methods (light microscopy) using splenic aspirates accompanied by typical clinical features such as; fever, weight loss, fatigue, anaemia, hepatomegaly, splenomegaly and presence of clinical response to anti-leishmanial treatment [1]. Control subjects were recruited from the same geographical region and matched for age, sex and ethnicity. Both, case and controls are Indo-European speakers and are socially classified as caste populations. The controls were healthy subjects, who have never been diagnosed with VL and did not show any family history of VL from the last three generations. The health status of the control subjects were examines with the help of local health authority and confirmed that they are healthy. Further, they also confirmed that the healthy subjects were also free from other infectious diseases (TB, filaria, malaria, etc.) of same geographical region. The mean age of all cases was 29.38 +/- 17.11, while controls ranged from 38.79 +/- 16.57 (Table 1). The male to female ratio in cases was 102:82 and in controls was 97:75 (Table 1). From each subject, we have collected 3–5.0 mL of peripheral blood samples in EDTA vacutainer, with informed written consent. Prior permission was also obtained from the district government authority. This study was approved by the Institutional Ethical Committee (IEC) of CSIR-Centre for Cellular and Molecular Biology, Hyderabad, India. In addition to case controls, a total of 1138 individuals from 34 ethnic populations belonging to different social (16 tribal population and 18 caste populations) and linguistic groups (Indo-European, Dravidian, and Austro-Asiatic); and inhabited in different geographical regions, were also included in this study). These samples were utilized from the DNA bank of CCMB (Centre for Cellular and Molecular Biology, Hyderabad).

### DNA isolation and IL10 genotyping

Genomic DNA was extracted from whole blood, using the protocol described previously [40]. Reference genomic sequence was retrieved from the Ensembl database [www.ensembl.org]. Primers for PCR and sequencing of five exons and exon-intron boundary were designed using Primer-BLAST (http://www.ncbi.nlm.nih.gov/tools/primer-blast) and synthesized commercially (Eurofins, India) (Table 2).

**Table 2:** Primer sequence, GC % and annealing temperature of *IL10* exons.

Primer	Sequence	GC%	Tm	Annealing temperature
IL10_1F	GGTTAGAGAAGGAGGAGCTCTAAGCA	50	60	
IL10_1R	GGCGCAGGAGGAGGGTTCTT	65	58	60
IL10_2F	GGGCATCAAAAAGACCGCATTTCAGT	46	58	
IL10_2R	TGTCCCTGCTGGTCTGTAGGA	57	56	58
IL10_3 & 4F	TCCCAGGGCCATGGAAGCAG	65	58	
IL10_3 & 4R	TGCACGTGTGGGTTCAGCCT	60	56	58
IL10_5AF	TCCCAGCGTGAGGGAGAACA	60	56	
IL10_5AR	GCGCCCGGCCTAGAACCAAA	65	58	58
IL10_5BF	GTTGAGCTGTTTTCCCTGACCTCCC	56	61	
IL10_5BR	GTCAGACAAGAGTCAACTGACACCAGA	48	60	61
IL10_5CF	CCTAAATTTGGTTCTAGGCCGGGCG	56	61	
IL10_5CR	TAGGGGGTAGCTGGCTTCCTTTCTC	56	61	61

We have amplified the target regions using primer pairs (Table 2) and an Emerald PCR master mix (TaKaRa). The reactions were carried out in an ABI GeneAmp PCR system 9700. The thermal cycling parameters used were as follows: initial denaturation at 95°C for 5 minutes, followed by 35 cycles of denaturation at 94°C for 1 minute, annealing for 30 seconds and elongation at 72°C for 1 minute (Table 2). PCR amplification was followed by Exo-SAP treatment (USB Corporation, USA), following manufacturer’s protocol. Exo-SAP treated amplicons were sequenced directly using BigDye terminator (v.3.1) cycle sequencing kit (Applied Biosystems, USA) on an ABI 3730XL DNA analyser. Sequence variations were identified by assembling DNA sequences with the reference sequence using AutoAssembler software (Applied Biosystems, USA). Variations obtained were validated and reconfirmed in a subset of samples by re-sequencing and visual confirmation of electropherograms.

### Statistical analysis

The target sample size was determined using PS software (Power and Sample Size Calculation Software Package, Vanderbilt University, Nashville, TN). The allele and genotype frequencies were calculated by simple gene counting method and Expectation Maximum (EM) algorithm. Hardy-Weinberg equilibrium and Chi-square tests were computed using PLINK software (Purcell et. al, 2007, options used:—assoc,—hwe 0.01). p value of < 0.05 was considered significant. Further, p value was corrected for multiple testing and adjusted as function of R base package [41]. Linkage disequilibrium (LD) analysis was performed using Haploview (v4.2). In addition, genetic models such as allelic, dominant and recessive were examined to evaluate the distribution of the genotype and allelic frequencies. *In-silico* methods were used to predict miRNA binding sites for wild and mutant (rs3024498) mRNA, using RegRNA online tool.

## Results

**Fig 2:**
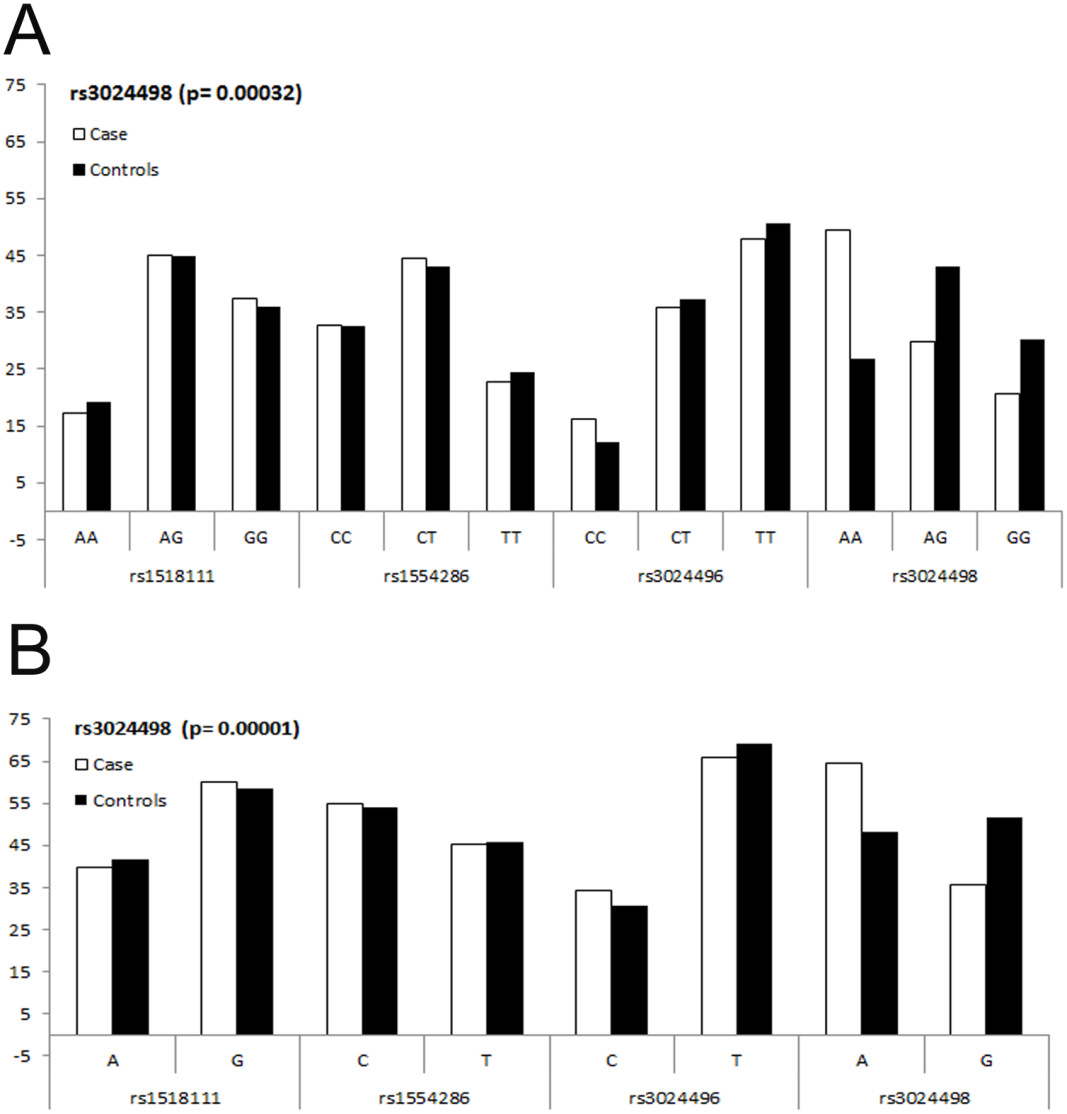
A. Distribution of genotype frequencies for*IL10* polymorphism, rs1518111 A>G; rs1554286 C>T; rs3024496 C>T and rs3024498 A>G in VL case and control subjects . B. Distribution of allele frequencies for *IL10* polymorphism, rs1518111 A>G; rs1554286 C>T; rs3024496 C>T and rs3024498 A>G in VL case and control subjects.

**Fig 3:**
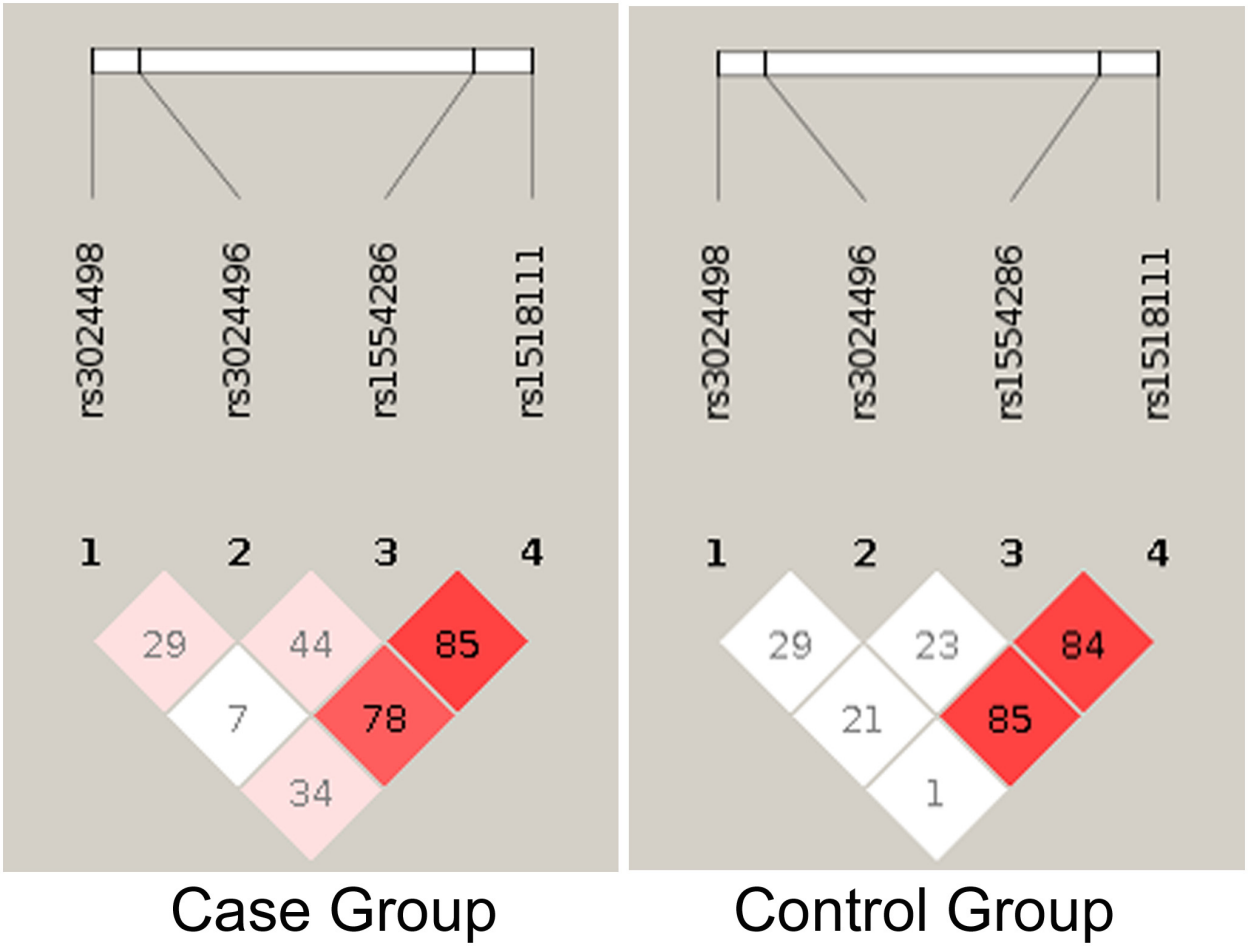
Linkage Disequilibrium (LD) of studied *IL-10* loci rs1518111 (2195 A>G, intron), rs1554286 (2607 C>T, intron), rs3024496 (4976 T>C, 3’ UTR), rs3024498 (5311 A>G, 3’ UTR) in VL case and control groups.

We estimated that 184 cases and 172 controls were required for each study to achieve 88% power, with 95% confidence, and 5% precision to detect a variable with an odds ratio of 2.0 (based on the assumption of 30% exposure among controls). Further, we have investigated the entire coding region of *IL10* in study subjects, with non-significant difference in age and gender distribution (Table 1), and found four variations (SNPs): rs1518111 (2195 A>G, intron 2; chr1:206945616), rs1554286 (2607 C>T, intron 3; chr1:206944233), rs3024496 (4976 T>C, 3’ UTR; chr1:206941864) and rs3024498 (5311 A>G, 3’ UTR; chr1:206941529) (Fig 1; Table 3). Of which, 5311 A>G in 3’ UTR is associated with visceral leishmaniasis by logistic regression analysis (Table 3). Suggesting that allele A is significantly associated with visceral leishmaniasis (χ^2^ = 18.87; p = 0.00001, OR = 0.515; CI = 0.382–0.696) (Table 3 and Fig 2A and 2B). However, the ‘G’ allele is significantly reducing risk of VL (Table 3, Fig 2B). The genotype distribution (%) of 5311 A>G in VL cases were 49.46 (AA), 29.89 (AG), 20.65 (GG), while in control they were 26.74 (AA), 43.02 (AG), 30.24 (GG) (Table 3 and Fig 2A). All the studied polymorphisms were in HWE for control subjects. Of these, GG (χ^2^ = 12.97; p = 0.00032, OR = 0.369; CI = 0.213–0.639) and AG (χ^2^ = 15.18; p = 0.00010, OR = .376; CI = 0.228–0.618) genotypes were significantly associated with protection against VL. Similarly, distributions of allele A was 64.40%, and G was 35.60% in cases; while in controls it was 48.26% (A), and 51.75% (G). Further, of the four SNPs studied, two (rs1554286 and rs1518111) were in LD in both cases (r2 = 0.78) and controls (r2 = 0.84) (Fig 3).

**Table 3:** Distribution of *IL10* genotype and alleles in cases and controls of visceral leishmaniasis.

SNP ID	Genotype	No. of Case (%)	No. of Controls (%)	OR	(95% CI)	χ^2^ Value	P- Value	Adjusted P-Value
rs1518111	AA	32(17.39)	33(19.19)	Ref				
	AG	83(45.11)	77(44.77)	1.112	0.624–1.979	0.13	0.71912	
	GG	69(37.50)	62(36.04)	1.148	0.633–2.081	0.21	0.64996	
	A	147(39.95)	143(41.57)	Ref				
	G	221(60.05)	201(58.43)	1.07	0.793–1.443	0.19	0.65938	
rs1554286	CC	60(32.60)	56(32.56)	Ref				
	CT	82(44.57)	74 (43.02)	1.034	0.639–1.673	0.02	0.89091	
	TT	42(22.83)	42(24.42)	0.933	0.532–1.637	0.06	0.8098	
	C	202(54.89)	186(54.07)	Ref				
	T	166(45.11)	158(45.93)	0.967	0.720–1.300	0.05	0.82589	
rs3024496	CC	30(16.30)	21(12.21)	Ref				
	CT	66(35.87)	64(37.21)	0.722	0.375–1.390	0.95	0.3287	
	TT	88(47.83)	87(50.58)	0.708	0.377–1.331	1.15	0.28277	
	C	126(34.24)	106(30.81)	Ref				
	T	242(65.76)	238(69.19)	0.855	0.625–1.171	0.95	0.32983	
rs3024498	AA	91(49.46)	46(26.74)	Ref				
	AG	55(29.89)	74(43.02)	0.376	0.228–0.618	15.18	0.0001	0.0004
	GG	38(20.65)	52(30.24)	0.369	0.213–0.639	12.97	0.00032	0.0018
	A	237(64.40)	166(48.26)	Ref				
	G	131(35.60)	178(51.74)	0.515	0.382–0.696	18.87	0.00001	0.00004

### *In-Silico* approach for functional validation of rs3024498

Bioinformatic analysis suggest four putative miRNA binding targets at rs3024498; miR-1236 (binding score 140, Δ G -9.20), miR-29b-2 (binding score 150, Δ G -13.80), miR-3192 (binding score 145, Δ G -23.90) and miR-4321 (binding score 140, Δ G -9.20) in wild type mRNA. In mutant, only three binding targets were found; miR-1236 (binding score 140, Δ G -9.20), miR-29b-2 (binding score 145, Δ G -11.20) and miR-3192 (binding score 141, Δ G -23.50).

### IL10 variation in diverse Indian populations

**Fig 4:**
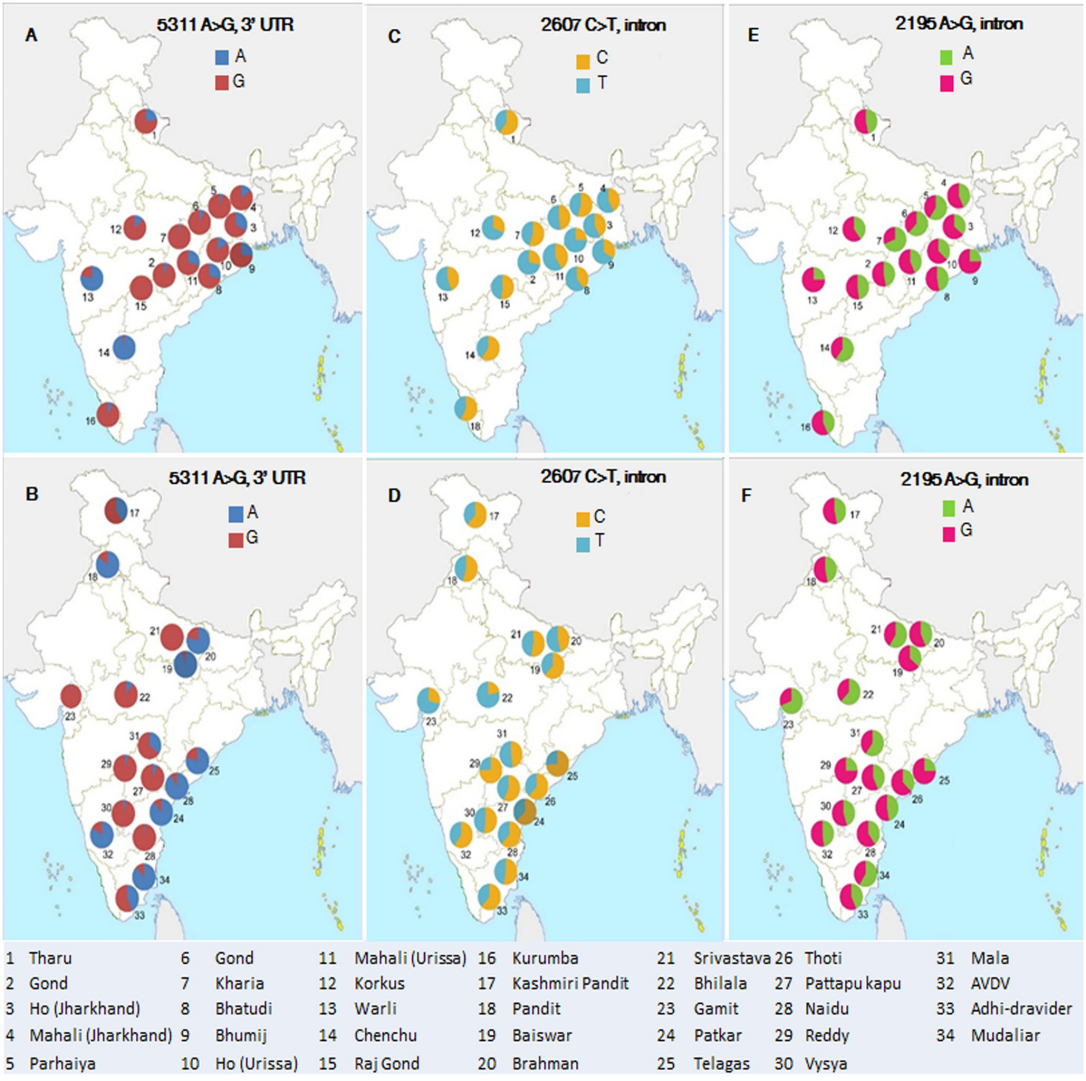
Geographical location and allele distribution of rs1518111 (2195 A>G, intron), rs1554286 (2607 C>T, intron) and rs3024498 (5311 A>G) polymorphism in tribe and caste population of India. Population no. 1 to 16 belongs to castes (Fig A, C and E). Population no. 17 to 34 belongs to tribes (Fig B, D and F).

Analysis of four IL10 SNPs, observed in case-control study was analysed in 1138 subjects belong to 34 populations across India revealed variable frequency. The allele data of rs3024498 shows that 24 out of 34 populations have higher frequency (>0.5) of G allele (mostly in tribal populations), and the remaining 10 populations showed high frequency of A allele (mostly in caste populations) (Table 4 and Fig 4).

**Table 4:** Population characteristics (sample size, language family and social designation) and geographical location of studied *IL-10* loci rs1518111 (2195 A>G, intron), rs1554286 (2607 C>T, intron), rs3024496 (4976 T>C, 3’ UTR), rs3024498 (5311 A>G, 3’ UTR) in Indian population.

S.No.	Population characteristics with demographic details						rs1518111		rs1554286		rs302396		rs302398	
	Population	Sample Size	Language family	Social Designation	State/territory	Latitude / longitude	A	G	C	T	C	T	A	G
1	Tharu	24	Indo-European	Tribal	Uttarakhand	29^°^23'N/79^°^30'E	0.46(22)	0.46(26)	0.60(29)	0.40(19)	0.17(8)	0.83(40)	0.23(11)	0.77(37)
2	Gond	24	Dravidian	Tribal	Jharkhand	22^°^67'N/86^°^33'E	0.46(22)	0.54(26)	0.48(23)	0.52(25)	0.23(11)	0.77(37)	0.06(3)	0.94(45)
3	Ho (Jharkhand)	44	Austro-Asiatic	Tribal	Jharkhand	23°35'N/85°33'E	0.58(30)	0.42(22)	0.42(22)	0.58(30)	0.10(5)	0.90(47)	0.33(17)	0.67(35)
4	Mahali (Jharkhand)	26	Austro-Asiatic	Tribal	Jharkhand	22°64'N/86°30'E	0.65(17)	0.35(9)	0.42(11)	0.58(15)	0.08(2)	0.92(24)	0.15(4)	0.85(22)
5	Parhaiya	13	Austro-Asiatic	Tribal	Jharkhand	22°60'N/86°36'E	0.52(43)	0.48(39)	0.55(45)	0.45(37)	0.24(20)	0.76(62)	0.05(4)	0.95(78)
6	Gond	41	Dravidian	Tribal	Chhattisgarh	19°87'N/81°60'E	0.68(56)	0.32(26)	0.28(23)	0.72(59)	0.13(11)	0.87(71)	0.07(6)	0.93(76)
7	Kharia	75	Austro-Asiatic	Tribal	Chhattisgarh	21°90'N/83°40'E	0.51(77)	0.49(73)	0.55(82)	0.45(68)	0.17(25)	0.83(125)	0.02(3)	0.98(147)
8	Bhatudi	38	Indo-European	Tribal	Urissa	21°90'N/86°70'E	0.59(45)	0.41(31)	0.39(30)	0.61(46)	0.09(7)	0.91(69)	0.29(22)	0.71(54)
9	Bhumij	41	Austro-Asiatic	Tribal	Urissa	21°93'N/86°73'E	0.68(56)	0.32(26)	0.34(28)	0.66(54)	0.22(18)	0.78(64)	0.26(21)	0.74(61)
10	Ho (Urissa)	40	Austro-Asiatic	Tribal	Urissa	21°91'N/86°74'E	0.74(59)	0.26(21)	0.25(20)	0.75(60)	0.19(15)	0.81(65)	0.16(13)	0.84(67)
11	Mahali (Urissa)	46	Austro-Asiatic	Tribal	Urissa	21°93'N/86°73'E	0.59(54)	0.41(38)	0.41(38)	0.59(54)	0.12(11)	0.88(81)	0.29(27)	0.71(65)
12	Korkus	42	Austro-Asiatic	Tribal	Madhya Pradesh	22°06'N/78°94'E	0.67(56)	0.33(28)	0.32(27)	0.68(57)	0.14(12)	0.86(72)	0.13(11)	0.87(73)
13	Warli	59	Indo-European	Tribal	Maharastra	19°17'N/72°95'E	0.50(59)	0.50(59)	0.44(52)	0.56(66)	0.18(21)	0.82(97)	0.79(93)	0.21(25)
14	Chenchu	20	Dravidian	Tribal	Andhra Pradesh	14°41'N/77°39'E	0.40(16)	0.60(24)	0.60(24)	0.40(16)	0.25(10)	0.75(30)	0.98(39)	0.03(1)
15	Raj Gond	28	Dravidian	Tribal	Andhra Pradesh	19°67'N/78°53'E	0.48(27)	0.52(29)	0.52(29)	0.48(27)	0.23(13)	0.77(43)	0.00(0)	1.00(56)
16	Kurumba	14	Dravidian	Tribal	Kerla	10°54'N/76°27'E	0.46(13)	0.54(15)	0.57(16)	0.43(12)	0.18(5)	0.82(23)	0.07(2)	0.93(26)
17	Kashmiri Pandit	42	Indo-European	Caste	Jammu & Kashmir	34°22'N/75°50'E	0.42(35)	0.42(49)	0.62(52)	0.38(32)	0.26(22)	0.74(62)	0.40(34)	0.60(50)
18	Pandit	33	Indo-European	Caste	Haryana	31°64'N/74°86'E	0.42(28)	0.42(38)	0.55(36)	0.45(30)	0.27(18)	0.73(48)	0.85(56)	0.15(10)
19	Baiswar	37	Indo-European	Caste	Uttar Pradesh	25°15'N/82°60'E	0.36(27)	0.64(47)	0.62(46)	0.38(28)	0.35(26)	0.65(48)	0.95(70)	0.05(4)
20	Brahman	36	Indo-European	Caste	Uttar Pradesh	25°73'N/82°68'E	0.43(31)	0.57(41)	0.46(33)	0.54(39)	0.40(29)	0.60(43)	0.79(57)	0.21(15)
21	Srivastava	17	Indo-European	Caste	Uttar Pradesh	25°15'N/82°60'E	0.59(20)	0.41(14)	0.53(18)	0.47(16)	0.24(8)	0.76(26)	0.00(0)	1.00(34)
22	Bhilala	41	Indo-European	Caste	Madhya Pradesh	22°60'N/75°30'E	0.63(55)	0.38(33)	0.22(19)	0.78(69)	0.13(11)	0.88(77)	0.10(9)	0.90(79)
23	Gamit	21	Indo-European	Caste	Gujrat	21°17'N/72°83'E	0.69(29)	0.31(13)	0.29(12)	0.71(30)	0.07(3)	0.93(39)	0.02(1)	0.98(41)
24	Patkar	19	Dravidian	Caste	Andhra Pradesh	15°80'N/78°10'E	0.47(18)	0.53(20)	0.63(24)	0.37(14)	0.13(5)	0.87(33)	0.89(34)	0.11(4)
25	Telagas	24	Dravidian	Caste	Andhra Pradesh	18°17'N/83°53'E	0.25(12)	0.75(36)	0.73(35)	0.27(13)	0.27(13)	0.73(35)	0.79(38)	0.21(10)
26	Thoti	34	Dravidian	Caste	Andhra Pradesh	16°51'N/80°64'E	0.37(25)	0.63(43)	0.62(42)	0.38(26)	0.16(11)	0.84(57)	0.91(62)	0.09(6)
27	Pattapu kapu	23	Dravidian	Caste	Andhra Pradesh	15°83'N/78°05'E	0.43(20)	0.57(26)	0.57(26)	0.43(20)	0.22(10)	0.78(36)	0.07(3)	0.93(43)
28	Naidu	21	Dravidian	Caste	Andhra Pradesh	13°20'N/79°11'E	0.40(17)	0.60(25)	0.62(26)	0.38(16)	0.29(12)	0.71(30)	0.02(1)	0.98(41)
29	Reddy	22	Dravidian	Caste	Andhra Pradesh	17°37'N/78°48'E	0.25(11)	0.75(33)	0.75(33)	0.25(11)	0.27(12)	0.73(32)	0.05(2)	0.95(42)
30	Vysya	55	Dravidian	Caste	Andhra Pradesh	14°31'N/77°44'E	0.45(49)	0.55(61)	0.52(57)	0.48(53)	0.23(25)	0.77(85)	0.04(4)	0.96(106)
31	Mala	20	Dravidian	Caste	Andhra Pradesh	18°68'N/78°10'E	0.60(24)	0.40(16)	0.48(19)	0.53(21)	0.20(8)	0.80(32)	0.35(14)	0.65(26)
32	AVDV (Karnataka)	25	Dravidian	Caste	Karnataka	12°58'N/77°35'E	0.48(24)	0.52(26)	0.60(30)	0.40(20)	0.30(15)	0.70(35)	0.84(42)	0.16(8)
33	Adhi-dravider	59	Dravidian	Caste	Tamilnadu	11°35'N/77°73'E	0.43(51)	0.57(67)	0.62(73)	0.38(45)	0.19(23)	0.81(95)	0.43(51)	0.57(67)
34	Mudaliar	34	Dravidian	Caste	Tamilnadu	12°92'N/79°13'E	0.59(40)	0.41(28)	0.53(36)	0.47(32)	0.16(11)	0.84(57)	0.88(60)	0.12(8)

The allele data of rs1554286 has shown that 20 out of 34 populations have higher frequency (>0.5) of T allele (mostly tribes), 11 populations have high frequency of C allele (mostly castes) and three populations have an equal frequency of T and C alleles (Table 4 and Fig 4).

The allelic data of rs1518111 has shown that 16 out of 34 populations have higher frequency (>0.5) of A allele (mostly in tribal populations) and the remaining 18 populations have high frequency of G allele (mostly in caste populations). The allelic data of rs3024496 shown that all the populations have higher frequency (>0.5) of T allele (Table 4 and Fig 4).

## Discussion

Indian populations are highly diverse due to strict endogamy and show variations in allele frequency in general [37]. Cytokine polymorphisms are usually associated with disease progression. Therefore, our aim was to investigate the IL10 functional variants that can modulate or alter serum IL10 levels, which may leads to either increased or decreased risk for infectious disease on whole, VL in particular. Study suggest that about 50% of IL10 production is determined by genetic factors, whereas the other half is accounted by additive environmental influence [42].

We have found a total of 4 SNPs in individuals inhabited in VL endemic regions, of the four SNPs, rs3024498 showed association with VL. Although this SNP has been found to be associated with active pulmonary tuberculosis, colorectal cancer, helminth infection, gastrointestinal stromal tumours and hepatitis C virus (HCV) clearance [19, 43–46], we are reporting for the first time its association with Indian VL. It has been shown that Indian VL patients exhibit higher levels of *IL10* in serum [10, 12, 31]. Since we found g.5311A (A allele of rs3024498) is associated with VL, we predict that this might be regulating *IL10* production, either through *cis*-regulatory mechanism or in association (haplotype) with other promoter SNPs. Several studies have established that the *IL10* gene expression is regulated by complex mechanisms [47–50]. It has also been demonstrated that the rs3024498 showed similar risk effects in HCV clearance [46].

Haploview analysis of our case-control study shows that the SNP in intron 2 (rs1518111) is in linkage disequilibrium (r2 = 0.78; Fig 3) with the SNP in intron 3 (rs1554286). Although these two SNPs were not associated with VL in our study, but they were reported with Behcet’s diseases in Han Chinese, Japan, Turkey and Korea populations [51, 52]. However, in India SNP rs1554286 has been found to be associated with leprosy [25].

India has one of the richest ethnic and linguistic diversity in South Asia and consists of more than four thousands of populations, including castes, tribes, primitive tribes and hunters and gatherers [37]. India is a home of several tribal pockets which represents 8.2% of the total population (2011 Census). The social structure of the Indian population is governed by the hierarchical caste system. We have demonstrated earlier that every single population in India is maintaining the endogamy for the last several thousand years, hence gained unique set of variations, which makes them genetically very distinct [37, 53]. In addition, we have also shown that the malaria susceptible allele is predominant in some populations, whereas others carry predominantly resistant allele [54, 55]. Having observed varying frequency of risk / resistant allele in different Indian populations, it would be worth to assess the frequency of all four IL10 polymorphisms, observed in the study, in 34 diverse Indian populations (1138 individuals, across India; Table 4). We have observed over representation of protective allele (G of rs3024498) for VL, and risk allele (T of rs1554286) for leprosy among the tribal populations across India, and *vice versa* for caste populations (Table 4, Fig 4). Interestingly, alleles A (rs1518111) that was found to be associated with Behcet’s disease in Han Chinese population [51] was observed predominantly among the tribes while caste populations have higher frequency (>0.50) of protected G allele.

Analysis of rs3024498 has shown that majority of tribal populations (14 out of 16), have high frequency of G allele (>0.50). However, we found only two tribal groups (Chenchu 0.02%; and Warli 0.21%) are with low frequency of G allele, suggesting that these two populations were under higher risk of VL. Nevertheless, there is no VL case reported neither in these two populations, nor in these regions (Andhra Pradesh and Maharastra respectively) (Table 4), which may be due to absence of Leishmania vector and non-favourable ecological conditions. Interestingly, although Chattisgarh, Jharkhand and Odisha states (tribes dominated states) were geographically closer to Bihar and majority of the populations inhabited in these states were genetically protected / frequency of risk alleles is lower (over- representation of G allele of SNP rs3024498). On the other hand, VL endemic / sporadic states (Bihar, West Bengal and Eastern part of Uttar Pradesh) were the caste dominated region and show over-representation of VL associated allele A. Our population data is in concordance with prevalence of VL in above or different states of India [56].

Comparison of the allele frequency of rs3024498 in different world populations showed that it varies across the populations. Interestingly, Gujarati Americans (American citizen with Indian ancestry) of HapMap Phase 3 (GIH) showed low frequency of MAF, compared to our study (Table 5). This is mainly due to their admixure with the local American (37). Therefore, data of GIH should not be used as a representative data for Indian population. Analysis of rs3024496 has shown its association with helminth disease and chlamydial infection in Brazil and African populations, respectively [42, 47], however it is not associated with VL in our study. Although three SNPs (rs1518111, rs1554286 and rs3024498), out of four showed significant difference between caste and tribal populations, the fourth SNP (rs3024496) did not show any significant difference between caste and tribal populations.

**Table 5:** Observed allele frequencies in the current study compared to reported allele frequencies in world populations.

Hap Map Database	Populations	rs3024498 (A/G)	
		A: freq (count)	G: freq (count)
This study	Case	0.356 (131)	0.644 (237)
	Control	0.5174 (178)	0.4826 (166)
Admix Indians	Gujarati Indians in Houston, Texas. (GIH)	0.07386 (13)	0.92614 (163)
Europeans	Toscans in Italy (TSI)	0.1989 (35)	0.8011 (141)
	Cental European (CEU)	0.2909 (64)	0.7091 (156)
Mexicans	Mexican ancestry in Los Angeles (MEX)	0.22 (22)	0.78 (78)
E-Asians	Han-Chinese-(HCB)	0 (0)	1 (164)
	Chinese in Metropolitan Denver, (CHD)	0.006024 (1)	0.993976 (165)
	Japanese in Tokyo (JPT)	0 (0)	1 (172)
Africans	Luhya in Webuye, Kenya (LWK)	0.1278 (23)	0.8722 (157)
	Maasai in Kinyawa, Kenya. (MKK)	0.1573 (45)	0.8427 (241)
	Yoruba (YRI)	0.0885 (20)	0.9115 (206)
	African ancestry in Southwest USA (ASW)	0.1146 (11)	0.8854 (85)

Earlier studies on rs1554286 suggest its role in leprosy in North India, where the associated T allele makes haplotype with promoter SNP [25]. This SNP has been found to be associated with Behcets disease and down regulates *IL10* expression in juvenile rheumatoid arthritis [29, 52]. Analysis of leprosy risk allele T (rs1554286) in different Indian populations showed that majority of tribal populations (10 out of 16), have higher frequency (>0.50%) of T allele compare to caste population (4 out of 18) (Table 4). Our data is in concordance with earlier fact that tribe dominated states (Jharkhand, Chattisgarh and Odisha) were among the high leprosy incidence state in India according to World Health Organization (www.who.int/lep/situation/india/states2006) (Table 6). Additionally, these states were also malaria endemic region [54]. Variation in rs1518111 was found to be associated with Behcet’s disease in Han Chinese, Japan, Turkey and Korea populations [51, 52]. Allele wise data indicate that majority of tribal populations (11 out of 16) were showing high frequency (>0.50%) of A allele, while majority of caste populations (13 out of 18) shows high frequency of G allele (Table 4). Since study on Han Chinese population shows G allele as a protective allele, so we can conclude that castes population were resistance for Behcet’s disease compare to tribes. Our data is in concordance to the fact that Behcet’s disease is very rare in India due to caste dominated population of India (2011 Census).

**Table 6:** Prevalence (yearly in millions) of different infectious disease (Visceral Leishmaniasis, Leprosy, Tuberculosis Malaria and Filaria) in worldwide and Indian region.

Diseases	Prevalence in World	Prevalence in India	Disease burden by region (India-IN / south east asia-SEA)	Reference
Visceral Leishmaniasis	~ 0.2–0.4	~ 0.28	~67% (SEA)	WHO; (5,38,39,56)
Leprosy	~0.25	~0.13	~67 (SEA)	WHO; (5,62)
Tuberculosis	~0.88	~0.22	~25% (IN)	WHO; (5,63)
Malaria	~207	~0.2	~77% of south east asia	WHO; (5,54,55,61)
Filaria	~120	~21	~26%	WHO; (5,64,65)

Furthermore, Haploview analysis of all four SNPs in 34 diverse Indian populations suggests that the LD varies from strong to moderate. The majority of the populations analysed (29 out of 34), showed LD between rs1518111 and rs1554286 (r2 >0.5).

Several studies established IL10 as important anti-inflammatory cytokine, which modulate the VL susceptibility and resistance via Th2/T regulatory responses and considered it as a master regulator of immunity (reviewed in [9]). Earlier genetic study shows role of IL10 polymorphisms in VL, CL and PKDL in different world populations (Iran, Brazil and Sudan) [34–36]. In India various immunological studies in the same endemic region of Bihar, as the present study region, have demonstrated that VL patients have higher level of sera IL10, which is a key regulatory cytokine, involved in inhibition of parasite clearance [10, 32, 33]. Interestingly, our genetic study on the same ethnic populations, showed association of IL10 variation with VL (rs3024498; p = 0.00001). Since the same SNP (rs3024498) along with promoter SNP, was known to involve in phenotype regulation in other population [46], further analysis of promoter region will help in understanding whether the SNP-rs3024498 alone or in combination with any promoter SNP leads to VL risk / severity. Additionally, absence of miR-4321 binding sites and change of binding scores and free energy (miR-29b-2* and miR-3192) in mutant-rs3024498 (A allele) suggest that this SNP might be dis-regulating the gene expression through improper miRNA binding, further affecting IL10 production and downstream functions of IL10. It is well established fact that genetic variants at miR binding sites are functional and important contributors to phenotype and diseases variation [57–60]. Presence of miR binding sites make this SNP relevant for further functional research. Since, diverse Indian populations were showing different frequency of risk alleles [54–55]. Therefore, we have to consider many populations or at least representative populations from different social and linguistic groups to assess the genetic basis of disease. India is one of major foci of VL, malaria, leprosy, tuberculosis and filarial infectious diseases however, presence of other less reported infectious disease in the region, feature a need for further research in this regard (Table 6) [56, 61–67]. In above context, this study provides valuable information on IL10 variation in Indian populations with disease perspective and demonstrate *IL10* association with VL. Finally, identification of high-risk individuals / populations through genetic analysis will increase our understanding of the genetic basis of VL and to gain better insight in to the pathological basis of the severity of the disease. Thus, further, functional and replication study from other regions would support to conclude our findings.

## Conclusion

In conclusion, we have found a variant g.5311A in *IL10*, which is associated with Indian VL. Further, this comprehensive study on *IL10* in Indian populations have shown variable frequency of the disease associated variant in different populations, which is in concordance with our earlier findings that different social and linguistic populations of India have different genetic composition that determine the susceptibility or resistance or severity of the disease. Our finding has potential medical implications and this information can be used for generating data on the neglected diseases and would help in management and forecast of the severity of disease.

## References

[1] Sundar S, Benjamin B. Diagnosis and treatment of Indian visceral leishmaniasis. J Assoc Physicians India. 2003;51:(7): 195–201. 12725267

[2] Desjeux P. Leishmaniasis. Public health aspects and control. Clin Dermatol. 1996;14(5):417–23. 8889319 10.1016/0738-081x(96)00057-0

[3] Hasker E, Kansal S, Malaviya P, Gidwani K, Picado A, Singh RP, et al Latent infection with Leishmania donovani in highly endemic villages in Bihar, India. PLoS Negl Trop Dis. 2013;7(2):e2053 10.1371/journal.pntd.0002053 23459501 PMC3573094

[4] Dinesh DS, Das ML, Picado A, Roy L, Rijal S, Singh SP, et al Insecticide susceptibility of Phlebotomus argentipes in visceral leishmaniasis endemic districts in India and Nepal. PLoS Negl Trop Dis. 2010;4(10):e859 10.1371/journal.pntd.0000859 21049013 PMC2964302

[5] Hotez PJ, Remme JH, Buss P, Alleyne G, Morel C, Breman JG. Combating tropical infectious diseases: report of the Disease Control Priorities in Developing Countries Project. Clin Infect Dis. 2004;38(6):871–8. 14999633 10.1086/382077

[6] Thakur CP. Socio-economics of visceral leishmaniasis in Bihar (India). Trans R Soc Trop Med Hyg. 2000;94(2):156–7. 10897353 10.1016/s0035-9203(00)90255-4

[7] Bora D. Epidemiology of visceral leishmaniasis in India. Natl Med J India. 1999;12(2):62–8. 10416321

[8] Fakiola M, Strange A, Cordell HJ, Miller EN, Pirinen M, Su Z, et al Common variants in the HLA-DRB1-HLA-DQA1 HLA class II region are associated with susceptibility to visceral leishmaniasis. Nat Genet. 2013;45(2):208–13. 10.1038/ng.2518 23291585 PMC3664012

[9] Couper KN, Blount DG, Riley EM. IL-10: the master regulator of immunity to infection. J Immunol. 2008;180(9):5771–7. 18424693 10.4049/jimmunol.180.9.5771

[10] Nylen S, Maurya R, Eidsmo L, Manandhar KD, Sundar S, Sacks D. Splenic accumulation of IL-10 mRNA in T cells distinct from CD4+CD25+ (Foxp3) regulatory T cells in human visceral leishmaniasis. J Exp Med. 2007;204(4):805–17. 17389235 10.1084/jem.20061141 PMC2118563

[11] Saraiva M, O'Garra A. The regulation of IL-10 production by immune cells. Nat Rev Immunol. 2010;10(3):170–81. 10.1038/nri2711 20154735

[12] Nylen S, Sacks D. Interleukin-10 and the pathogenesis of human visceral leishmaniasis. Trends Immunol. 2007;28(9):378–84. 17689290 10.1016/j.it.2007.07.004

[13] Mosser DM, Zhang X. Interleukin-10: new perspectives on an old cytokine. Immunol Rev. 2008;226:205–18. 10.1111/j.1600-065X.2008.00706.x 19161426 PMC2724982

[14] Sabat R, Grutz G, Warszawska K, Kirsch S, Witte E, Wolk K, et al Biology of interleukin-10. Cytokine Growth Factor Rev. 2010;21(5):331–44. 10.1016/j.cytogfr.2010.09.002 21115385

[15] Moore KW, de Waal Malefyt R, Coffman RL, O'Garra A. Interleukin-10 and the interleukin-10 receptor. Annu Rev Immunol. 2001;19:683–765. 11244051 10.1146/annurev.immunol.19.1.683

[16] Cummings HE, Tuladhar R, Satoskar AR. Cytokines and their STATs in cutaneous and visceral leishmaniasis. J Biomed Biotechnol. 2010;2010:294389 10.1155/2010/294389 20300429 PMC2840379

[17] Vouldoukis I, Becherel PA, Riveros-Moreno V, Arock M, da Silva O, Debre P, et al Interleukin-10 and interleukin-4 inhibit intracellular killing of Leishmania infantum and Leishmania major by human macrophages by decreasing nitric oxide generation. Eur J Immunol. 1997;27(4):860–5. 9130636 10.1002/eji.1830270409

[18] Miyazoe S, Hamasaki K, Nakata K, Kajiya Y, Kitajima K, Nakao K, et al Influence of interleukin-10 gene promoter polymorphisms on disease progression in patients chronically infected with hepatitis B virus. Am J Gastroenterol. 2002;97(8):2086–92. 12190181 10.1111/j.1572-0241.2002.05926.x

[19] Abhimanyu, Mangangcha IR, Jha P, Arora K, Mukerji M, Banavaliker JN, et al Differential serum cytokine levels are associated with cytokine gene polymorphisms in north Indians with active pulmonary tuberculosis. Infect Genet Evol. 2011;11(5):1015–22. 10.1016/j.meegid.2011.03.017 21463712

[20] Haanpaa M, Nurmikko T, Hurme M. Polymorphism of the IL-10 gene is associated with susceptibility to herpes zoster. Scand J Infect Dis. 2002;34(2):112–4. 21. 11928840 10.1080/00365540110077218

[21] Howell WM, Turner SJ, Bateman AC, Theaker JM. IL-10 promoter polymorphisms influence tumour development in cutaneous malignant melanoma. Genes Immun. 2001;2(1):25–31. 11294564 10.1038/sj.gene.6363726

[22] Alamartine E, Berthoux P, Mariat C, Cambazard F, Berthoux F. Interleukin-10 promoter polymorphisms and susceptibility to skin squamous cell carcinoma after renal transplantation. J Invest Dermatol. 2003;120(1):99–103. 12535204 10.1046/j.1523-1747.2003.12016.x

[23] Tagore A, Gonsalkorale WM, Pravica V, Hajeer AH, McMahon R, Whorwell PJ, et al Interleukin-10 (IL-10) genotypes in inflammatory bowel disease. Tissue Antigens. 1999;54(4):386–90. 10551422 10.1034/j.1399-0039.1999.540408.x

[24] Shin HD, Winkler C, Stephens JC, Bream J, Young H, Goedert JJ, et al Genetic restriction of HIV-1 pathogenesis to AIDS by promoter alleles of IL10. Proc Natl Acad Sci U S A. 2000;97(26):14467–72. 11121048 10.1073/pnas.97.26.14467 PMC18942

[25] Aggarwal S, Ali S, Chopra R, Srivastava A, Kalaiarasan P, Malhotra D, et al Genetic variations and interactions in anti-inflammatory cytokine pathway genes in the outcome of leprosy: a study conducted on a MassARRAY platform. J Infect Dis. 2011;204(8):1264–73. 10.1093/infdis/jir516 21917900

[26] Lokossou AG, Dechavanne C, Bouraima A, Courtin D, Le Port A, Ladekpo R, et al Association of IL-4 and IL-10 maternal haplotypes with immune responses to P. falciparum in mothers and newborns. BMC Infect Dis. 2013;13:215 10.1186/1471-2334-13-215 23668806 PMC3679728

[27] Mege JL, Meghari S, Honstettre A, Capo C, Raoult D. The two faces of interleukin 10 in human infectious diseases. Lancet Infect Dis. 2006;6(9):557–69. 16931407 10.1016/S1473-3099(06)70577-1

[28] Metenou S, Dembele B, Konate S, Dolo H, Coulibaly SY, Coulibaly YI, et al Patent filarial infection modulates malaria-specific type 1 cytokine responses in an IL-10-dependent manner in a filaria/malaria-coinfected population. J Immunol. 2009;183(2):916–24. 10.4049/jimmunol.0900257 19561105 PMC2789677

[29] Crawley E, Kay R, Sillibourne J, Patel P, Hutchinson I, Woo P. Polymorphic haplotypes of the interleukin-10 5' flanking region determine variable interleukin-10 transcription and are associated with particular phenotypes of juvenile rheumatoid arthritis. Arthritis Rheum. 1999;42(6):1101–8. 10366102 10.1002/1529-0131(199906)42:6<1101::AID-ANR6>3.0.CO;2-Y

[30] Hoh J, Ott J. Mathematical multi-locus approaches to localizing complex human trait genes. Nat Rev Genet. 2003;4(9):701–9. 12951571 10.1038/nrg1155

[31] Ansari NA, Saluja S, Salotra P. Elevated levels of interferon-gamma, interleukin-10, and interleukin-6 during active disease in Indian kala azar. Clin Immunol. 2006;119(3):339–45. 16540374 10.1016/j.clim.2006.01.017

[32] Gautam S, Kumar R, Maurya R, Nylen S, Ansari N, Rai M, et al IL-10 neutralization promotes parasite clearance in splenic aspirate cells from patients with visceral leishmaniasis. J Infect Dis. 2011;204(7):1134–7. 10.1093/infdis/jir461 21881130 PMC3164427

[33] Singh OP, Stober CB, Singh AK, Blackwell JM, Sundar S. Cytokine responses to novel antigens in an Indian population living in an area endemic for visceral leishmaniasis. PLoS Negl Trop Dis. 2012;6(10):e1874 10.1371/journal.pntd.0001874 23150744 PMC3493615

[34] Hajilooi M, Sardarian K, Dadmanesh M, Matini M, Lotfi P, Bazmani A, et al Is the IL-10–819 polymorphism associated with visceral leishmaniasis? Inflammation. 2013;36(6):1513–8. 10.1007/s10753-013-9693-0 23912644

[35] Salhi A, Rodrigues V Jr, Santoro F, Dessein H, Romano A, Castellano LR, et al Immunological and genetic evidence for a crucial role of IL-10 in cutaneous lesions in humans infected with Leishmania braziliensis. J Immunol. 2008;180(9):6139–48. 18424735 10.4049/jimmunol.180.9.6139

[36] Farouk S, Salih MA, Musa AM, Blackwell JM, Miller EN, Khalil EA, et al Interleukin 10 gene polymorphisms and development of post kala-azar dermal leishmaniasis in a selected sudanese population. Public Health Genomics. 2010;13(6):362–7. 10.1159/000272457 20051670 PMC2951725

[37] Reich D, Thangaraj K, Patterson N, Price AL, Singh L. Reconstructing Indian population history. Nature. 2009;461(7263):489–94. 10.1038/nature08365 19779445 PMC2842210

[38] Singh SP, Reddy DC, Mishra RN, Sundar S. Knowledge, attitude, and practices related to Kala-azar in a rural area of Bihar state, India. Am J Trop Med Hyg. 2006;75(3):505–8. 16968930

[39] Singh SP, Reddy DC, Rai M, Sundar S. Serious underreporting of visceral leishmaniasis through passive case reporting in Bihar, India. Trop Med Int Health. 2006;11(6):899–905. 16772012 10.1111/j.1365-3156.2006.01647.x

[40] Thangaraj K, Joshi MB, Reddy AG, Gupta NJ, Chakravarty B, Singh L. CAG repeat expansion in the androgen receptor gene is not associated with male infertility in Indian populations. J Androl. 2002;23(6):815–8. 12399527

[41] Team RDC. R: a language and environment for statistical computing. Vienna (Austria): R Foundation for Statistical Computing 2009.

[42] Reuss E, Fimmers R, Kruger A, Becker C, Rittner C, Hohler T. Differential regulation of interleukin-10 production by genetic and environmental factors—a twin study. Genes Immun. 2002;3(7):407–13. 12424622 10.1038/sj.gene.6363920

[43] Tsilidis KK, Helzlsouer KJ, Smith MW, Grinberg V, Hoffman-Bolton J, Clipp SL, et al Association of common polymorphisms in IL10, and in other genes related to inflammatory response and obesity with colorectal cancer. Cancer Causes Control. 2009;20(9):1739–51. 10.1007/s10552-009-9427-7 19760027 PMC4119174

[44] Figueiredo CA, Barreto ML, Alcantara-Neves NM, Rodrigues LC, Cooper PJ, Cruz AA, et al Coassociations between IL10 polymorphisms, IL-10 production, helminth infection, and asthma/wheeze in an urban tropical population in Brazil. J Allergy Clin Immunol. 2013;131(6):1683–90. 10.1016/j.jaci.2012.10.043 23273955 PMC5017514

[45] O'Brien KM, Orlow I, Antonescu CR, Ballman K, McCall L, Dematteo R, et al Gastrointestinal stromal tumors: a case-only analysis of single nucleotide polymorphisms and somatic mutations. Clin Sarcoma Res. 2013;3(1):12 10.1186/2045-3329-3-12 24159917 PMC3827940

[46] Oleksyk TK, Thio CL, Truelove AL, Goedert JJ, Donfield SM, Kirk GD, et al Single nucleotide polymorphisms and haplotypes in the IL10 region associated with HCV clearance. Genes Immun. 2005;6(4):347–57. 15815689 10.1038/sj.gene.6364188

[47] Gibson AW, Edberg JC, Wu J, Westendorp RG, Huizinga TW, Kimberly RP. Novel single nucleotide polymorphisms in the distal IL-10 promoter affect IL-10 production and enhance the risk of systemic lupus erythematosus. J Immunol. 2001;166(6):3915–22. 11238636 10.4049/jimmunol.166.6.3915

[48] Powell MJ, Thompson SA, Tone Y, Waldmann H, Tone M. Posttranscriptional regulation of IL-10 gene expression through sequences in the 3'-untranslated region. J Immunol. 2000;165(1):292–6. 10861064 10.4049/jimmunol.165.1.292

[49] Natividad A, Holland MJ, Rockett KA, Forton J, Faal N, Joof HM, et al Susceptibility to sequelae of human ocular chlamydial infection associated with allelic variation in IL10 cis-regulation. Hum Mol Genet. 2008;17(2):323–9. 17947295 10.1093/hmg/ddm310

[50] Suarez A, Castro P, Alonso R, Mozo L, Gutierrez C. Interindividual variations in constitutive interleukin-10 messenger RNA and protein levels and their association with genetic polymorphisms. Transplantation. 2003;75(5):711–7. 12640314 10.1097/01.TP.0000055216.19866.9A

[51] Wu Z, Zheng W, Xu J, Sun F, Chen H, Li P, et al IL10 polymorphisms associated with Behcet's disease in Chinese Han. Hum Immunol. 2014;75(3):271–6. 10.1016/j.humimm.2013.11.009 24269690

[52] Mizuki N, Meguro A, Ota M, Ohno S, Shiota T, Kawagoe T, et al Genome-wide association studies identify IL23R-IL12RB2 and IL10 as Behcet's disease susceptibility loci. Nat Genet. 2010;42(8):703–6. 10.1038/ng.624 20622879

[53] Moorjani P, Thangaraj K, Patterson N, Lipson M, Loh PR, Govindaraj P, et al Genetic evidence for recent population mixture in India. Am J Hum Genet. 2013;93(3):422–38. 10.1016/j.ajhg.2013.07.006 23932107 PMC3769933

[54] Jha AN, Singh VK, Kumari N, Singh A, Antony J, van Tong H, et al IL-4 haplotype -590T, -34T and intron-3 VNTR R2 is associated with reduced malaria risk among ancestral indian tribal populations. PLoS One. 2012;7(10):e48136 10.1371/journal.pone.0048136 23110190 PMC3480467

[55] Jha AN, Sundaravadivel P, Singh VK, Pati SS, Patra PK, Kremsner PG, et al MBL2 variations and malaria susceptibility in Indian populations. Infect Immun. 2014;82(1):52–61. 10.1128/IAI.01041-13 24126531 PMC3911836

[56] Alvar J, Velez ID, Bern C, Herrero M, Desjeux P, Cano J, et al Leishmaniasis worldwide and global estimates of its incidence. PLoS One. 2012;7(5):e35671 10.1371/journal.pone.0035671 22693548 PMC3365071

[57] Zhang J, Cheng Y, Cui W, Li M, Li B, Guo L. MicroRNA-155 modulates Th1 and Th17 cell differentiation and is associated with multiple sclerosis and experimental autoimmune encephalomyelitis. J Neuroimmunol. 2014;266(1–2):56–63. 10.1016/j.jneuroim.2013.11.009 24332164

[58] Friedman JM, Jones PA. MicroRNAs: critical mediators of differentiation, development and disease. Swiss Med Wkly. 2009;139(33–34):466–72. doi: smw-12754 19705306 10.4414/smw.2009.12794 PMC2854010

[59] Lewis BP, Burge CB, Bartel DP. Conserved seed pairing, often flanked by adenosines, indicates that thousands of human genes are microRNA targets. Cell. 2005;120(1):15–20. 15652477 10.1016/j.cell.2004.12.035

[60] Krek A, Grun D, Poy MN, Wolf R, Rosenberg L, Epstein EJ, et al Combinatorial microRNA target predictions. Nat Genet. 2005;37(5):495–500. 15806104 10.1038/ng1536

[61] Kumar A, Valecha N, Jain T, Dash AP. Burden of malaria in India: retrospective and prospective view. Am J Trop Med Hyg. 2007;77(6 Suppl):69–78. 18165477

[62] Leprosy update, 2011. Wkly Epidemiol Rec. 2011;86(36):389–99. 21887885

[63] WHO. National Filariasis Control Programme in India and New Strategies for Its Control. (Cited 2005 May14). Available from http://www.who.int.india/communicablediseasessurveillances/filariasis.html

[64] WHO Global tuberculosis report 2012. Geneva2012.

[65] ICMR. Prospects of eliminating lymphatic filariasis in India. ICMR, 2002.

[66] Agrawal MC, Rao VG. Indian schistosomes: a need for further investigations. J Parasitol Res. 2011;2011:250868 10.1155/2011/250868 22132307 PMC3205607

[67] Agrawal M. The Schistosomes Schistosomes and Schistosomiasis in South Asia: Springer India; 2012.

